# The Nursing Supplement

**Published:** 1889-10-19

**Authors:** 


### The Nursing Mirror.

All Contributions for this Supplement should be addressed to the Editor, The Hospital, 139-140, Salisbury Court, Fleet Street, London, E.C., and should have the word "Nursing" plainly written in left-hand top corner of the envelope.

### En Passant.

NATIONAL PENSION FUND.—An interesting account of the second general meeting will be found on pages 47 and 48 of to-day's issue.

MISS ALCOTT.—Many of our readers will be glad to learn that the Life and Letters of Louisa M. Alcott have just been published. During the American Civil War the beloved authoress of "Little Women" acted for some time as a nurse, and though she used up her experiences in "Something to Do" and "Work," the actual place and nature of her ministrations were hidden under a veil of fiction. Now we hope to have a full and true report of Miss Alcott's nursing labours.

ADDENBROOKE'S HOSPITAL.—The following recommendation has been adopted by the committee of Addenbrooke's: "That the court sanction the erection of an additional building adjoining the south end of the nurses' dormitory (24ft.) not exceeding in length 30ft., and at a cost not exceeding £550, provided that £250 of this sum of £550 be given by the committees of the Bazaar and Alice Fisher Funds, such additional building to be used for the accommodation of the night superintendent, and for a ward for sick nurses and probationers."

THE HOTEL DIEU.—Being in Paris lately we remembered the reports about the nursing of the hospitals there—how the Republic had turned out all the Sisters of Charity, and then had to take them back again—and we strolled into the Hotel Dieu to see what foundation there was for these reports. Apparently there was some truth in them both, for over each ward a sister in full canonicals reigned supreme, and under her were certain dowdy middle-aged women who acted as nurses. These last wore black dresses and white aprons, and ugly little caps; there was no smartness nor neatness about them, and the sister's dress, though clean in the extreme, was horribly clumsy and liable to harbour dust and germs. The wards also lacked those little niceties—plants, flowers, pictures, etc.—which make our own wards so cheerful, and altogether we felt glad our work did not lie in a Parisian hospital.

PRACTICAL JOKE ON A MATRON.—An extraordinary story comes from the Infirmary Committee of the Salford Board of Guardians. On the 1st inst., when Miss Huguenin, the matron, was about to retire for the night, she found that a nest of rats had been placed in her bed. She was much alarmed, and spent the night seated on a chair in her bedroom. The act caused great indignation among the members of the committee, and they passed a resolution recommending that a letter be written to each of the officers and servants of the infirmary, excepting the medical officers, steward, and chaplain, stating that if information was not given to the committee by Wednesday next as to the person or persons who were guilty of placing the rats in the matron's bed they would all be dismissed with a month's notice from the service of the guardians. The resolution was confirmed with only two dissentients. It is nice to see the sorrows of a matron meet with such sympathy, but it is a pity to make such a fuss about a practical joke. It takes more than a nest of rats to frighten most infirmary matrons.

SHORT ITEMS.—The new nurses' home connected with the Queen's Hospital, Birmingham, has been formally opened. It contains 26 bedrooms and a sitting-room.—At a concert at Birmingham General Hospital last week Miss L. Taylor, one of the nursing staff, sang twice with great effect.—Miss Constance Newton, whose late interest in Jaffa is well known, has volunteered to go to Cyprus to organise a nursing staff at Nicosia.—The East of Scotland Tactical Society will give courses of lectures to nurses in Edinburgh this winter.—Miss Florence Nightingale holds shares in the co-operative society formed amongst bass dressers.

GRIMSBY HOSPITAL.—At the annual meeting, on October 7th, of governors of this hospital, it was stated that the system of home nurses, which was inaugurated during the past year, has been fully appreciated by the public who have availed themselves of the advantages of the trained nurses. During the year ending June 30th, 1889, 326 cases have been admitted into the wards, 7,750 outdoor patients have been attended to, and 3,120 visits have been made by the nurses to the homes of the patients. The Chairman also said the thanks of the committee were due to the medical staff and nurses for their great help.

LADIES AS DISTRICT NURSES.—The only position in the nursing world where a lady is perhaps rather at a discount, just because she is a lady, is in district nursing. A lady has more difficulty in getting *en rapport* with the poor, and appreciating their trials. Some institutions, where ladies only are trained, simply clean their patients out of house and home, and by over strictness get themselves disliked. Still, a lady need not necessarily make herself obnoxious, though it is far harder for her to leave things alone when they are not according to her ideas. The East London Nursing Society has hitherto confined itself to respectable women who were well trained, but Miss Brancher, the new matron, has introduced the novelty of having a lady friend to live with her and act as nurse. We watch the experiment with interest.

EXHIBITION OF NURSING UNIFORMS.—We have had many new entries for the Doll Show, including the Metropolitan Hospital, Warneford Hospital, Liverpool Children's Infirmary, Northwich Victoria Infirmary, and others. Still there are several large hospitals we should like to see represented from which no candidates have yet appeared. For instance, we should like some enterprising nurses from Manchester Royal, Salisbury, Aberdeen, Charing Cross, Gloucester, Bristol, Exeter, Macclesfield, Hereford, Hertford, Bootle Borough, Jenny Lind Infirmary, St. John the Divine, Nottingham, Addenbrooke's, Jaffray Hospital, Worcester, Bradford, Scarborough, and many other places, to send in their names. The exhibition will be so much more interesting if all uniforms are included. "Patience" asks if the public will be given a chance of seeing the show. We think it would be a good plan to charge all those not connected with nursing a small fee for admittance, and give the money thus received in extra prizes. In the "Queries" last week it was wrongly stated that dolls would be received till October 31st. This is a mistake. Names will be received till October 31st, dolls will be received between the 23rd and 30th of November. Next week we propose to reproduce all the conditions.

### Notes from Australia.

*(By Our Own Correspondent.)*

Melbourne, Sept. 1.

Mr. Williams, the secretary to the Melbourne Hospital, has just returned from his trip to England and America, and says he has been profoundly impressed with the marvellous progress of your whole nursing system. He says: "'Sairey Gamp is dead. You do not find her in the meanest hospital of the poorest workhouse in England. In her stead a lady has arisen by every sick bed—a tender, competent, ministering angel. Everywhere I find that the true importance of the nurse's position is recognised. She is chosen now from a good stock—all her antecedents are enquired into. She is trained there carefully in all the duties of her position. And her education finished, she is admitted to the work, which is arduous and painful, and treated always as one from whom much is required, and therefore to whom much should be rendered. Her status is that of a lady. She has a private room, and meets her associates in well-appointed parlours or drawing-rooms. No menial work is required of her, and she is regarded by the doctor as an assistant and associate. It is a marvellous, as a welcome change. It works for good in all ways. I do sincerely hope that it may be imitated or adopted here, for though our system is a little better now, I must say that a while ago it was atrociously and scandalously bad." You will see by this that it is at last dawning upon our good citizens that a trained nurse is better than an untrained one. Yet the new matron, Mrs. Miller, not long appointed, had no training, and Mr. Williams had better start English notions at once at his hospital.

Mr. Christie Murray, the noted novelist, refused several social invitations in order to visit the Melbourne slums. He was taken to several opium dens, where larrikins, and half-castes, and white women were all lying prostrate amidst the poisonous fumes. He was taken to a gambling den, where a crowd of gloomy, eager men crowded about a counter where a man with a stolid face sold tickets. An hour later this man "draws his bank" and pays the winners. What chiefly struck Christie Murray was the lack of anything like the want and desperate poverty of whole districts in London. I have been several times to the slums lately with a Bible-woman, who has worked in them for nine years. I do not think she would agree that "there is no poverty properly so called," though there is not the extreme misery of the London poor. There are many cases where poverty has come through over speculation, misfortune after misfortune, a want of adaption to the surroundings of a new country, till one is aghast at the state of men who have been in the colony thirty or forty years, who are now sixty or seventy-five years of age, with literally nothing to live on, yet have had splendid chances. Opium eating is a terrible evil among women as well as men. In the Chinese quarters there are several English women. They are never visible in the day time till three or four o'clock in the afternoon! One woman I know of will spend four or five shillings per day in opium when she can get it.

Dr. Shields reports an outbreak of diphtheria at Woodend. The disease had been spread in this way: the State school was cleaned out by a woman and her son, and the boy used to take the key and leave it at the parsonage, which was opposite. The clergyman's little daughter used to receive the key, and when the boy took diphtheria the girl contracted the disease also. The two first cases which occurred, and which seem to have infected the school, happened in the families of the local nightman and the overseer of the manure depot, both children being taken ill on the same day, so that it was almost certain that the disease had originated in connection with the nightsoil, and was carried to the school as a centre. The disease commenced with sore throat, and the local medical man declared it to be quinsy, or sore throat. It was difficult to get medical men to realise that an outbreak of diphtheria commenced in the first case with sore throat.

The Children's Hospital issues a good report, particularly with regard to the Convalescent Cottage at Brighton. A year ago very few people ever visited the small convalescents, but, thanks to a good example, the ladies of the neighbourhood now take it in turn to visit and play with the children, and to provide them with extra treats for tea. The water at Malmsbury and other places in the neighbourhood has had a very unpleasant taste and smell lately, owing to the presence of a minute plant called protococcus. It is said not to be injurious, but is certainly very unpleasant.

The reformatory at Coburg, where there are thirty-five girls at present, deserves some notice. The matron has the girls very well in hand, and they appear much attached to her. A sign of the favourable influence of those in charge of the reformatory lies in the fact that girls whose time had expired are frequently brought back to the matron by their mothers for further improvement, some return voluntarily while awaiting situations. By means of a useful book kept by the matron, and called the "After Career Book," a girl may be traced at any time, even though she may have left everything connected with the institution years behind.

The annual ball in aid of the funds of the Homœopathic Hospital has been held. As special efforts are at present being made to raise the sum necessary to claim £10,000 which has been conditionally promised by an unknown donor, the promoters were much pleased at the large attendance. His Excellency the Acting Governor, Sir William Robinson, had promised his patronage, but owing to his visit to Adelaide he was unable to attend. Lady Robinson and suite, however, were present, and were received by Sir Arthur Nicholson and the committee.

At the last meeting of the Alfred Hospital Committee the matron (Mrs. Strong), in accordance with the regulations, furnished a report of her observations with regard to the capabilities of the nurses who lately passed the preliminary examination conducted by the examiners of the Nurse Training School, Dr. Schlesinger and Dr. M. Barclay Thomson. The conduct and efficiency of the eight pupils who passed had been good, and there had been no suspensions. Of the number stated, three were considered suitable for hospital nursing; the remainder were thought to be more suitable for private nurses. The report was adopted.

It is reported that there have been forty-five cases of diphtheria during the past week, seven having proved fatal.

His Excellency the Acting Governor distributes the medallions and certificates to successful students of the St. John's Ambulance Association to-morrow night. Lady Robinson will also be present.

Mr. R. C. Norman, a member of the Charity Organisation Society, has written a letter to the *Argus* in which he says that during the year ending June 30, 1888, charitable relief was afforded in over 100,000 instances by the various philanthropic societies and institutions of Melbourne, at an aggregate cost of over £110,000 sterling. These figures do not include neglected or reformatory-school children, lunatics, or prisoners. Neither do they include persons in receipt of private or semi-private benevolence, nor the money so expended, though I have good reason for believing that both are very considerable. Surely statistics like these point to one or both of two conclusions—viz., either there is an appalling amount of poverty in the midst of all our wealth and vaunted prosperity, or relief is recklessly and, in many cases, unnecessarily bestowed. My experience leads me to believe that the latter is nearest the truth. Instead, therefore, of multiplying relieving agencies and making fresh appeals to the charitable, our first efforts should be to check this waste of charity by diverting its stream from the undeserving.

### Cottage Hospital Matrons.

### I.—THE TRAINING REQUIRED.

It has, I think, become a nearly universal custom that a cottage hospital of from twelve to twenty-five beds should have a thoroughly-trained matron or lady superintendent at its head, capable of undertaking the sole supervision of household and nursing departments, and of training her own probationers. Certainly, for thoroughness in the nursing department and comfort in the house, this system works far better than a ladies' committee, with as many different ideas on both points as there are ladies on the said committee. I believe this thoroughness and the general welfare of all concerned will amply repay any additional cost incurred, which cost may be reduced by taking paying probationers, an impossible course where no special head could be held responsible for their training. In addition, a system which I know to answer well in at least one hospital might be adopted—namely, the appointment, twice yearly, of two ladies, to provide the requisite furniture, etc., of every department, as it may require renewal; also of visitors to read to the patients at stated times.

Then arise the questions, What should be expected of such a matron, and how shall she be best trained to fill such a post.

To take the second question first. It is certainly best that such a matron should be a lady, as in all the large hospitals. It will be a great help to her if she has first learnt housekeeping and had supervision of servants at home, but if not, she certainly ought to acquire this in her training before attempting to fill this post.

She must have had a thorough hospital training, and have learnt all the duties of a nurse, including the necessity of obedience to the medical staff, and the minor duties of dressers. Anyone specially desirous of becoming a matron should to her training, if in a London hospital, add at least six months in a large provincial hospital, or, better still, in a cottage hospital, where dressers are few. She should be allowed opportunities for studying household management under the matron or housekeeper in such an establishment, with the understanding that she shall be taught all matters connected with the management of, and work in, a small hospital, and every particular of housekeeping, nursing, and the keeping of books, accounts, etc.

The duties, though at times affording ample leisure, are multifarious, and knowledge of what is required, and tact in carrying it out, are surely far better acquired by training before taking responsibility, than by the bitter experience gained from subsequent mistakes. Even if unable at once to procure the desired post, a lady so trained will have lost nothing if she take up work in another nursing department while she waits. Again, a lady may train for this post entirely in a cottage hospital, where her medical officers will take an interest in teaching her how cottage hospital nursing can best be done to their satisfaction and the patients' benefit. She may become thereby a very efficient matron, as Mr. Burdett has already suggested in his work on cottage hospitals. (Published by J. and A. Churchill.)

*(To be continued.)*

### Everybody's Opinion.

[*Correspondence on all subjects is invited, but we cannot in any way be responsible for the opinions expressed by our correspondents. No communications can be entertained if the name and address of the correspondent is not given, or unless one side of the paper only be written on.*]

### REGISTRATION OF MIDWIVES.

Elinor Agnes Bedingfield writes: May I call your attention to a leading article in the current number of a contemporary (October 3) which seems to be a protest against the work of the Matrons' Aid Society, because it has pushed the Bill for the Licensing and Registration of Midwives by Act of Parliament so far as to have had notice given that the Bill will be introduced next session. I know from past experience that it is all but hopeless to get anything inserted in the paper in question, unless the matter is entirely in accord with the ideas of that paper, or the wire-pullers of the British Nursing Association. May I therefore ask insertion of a few remarks on the leader in question. About a year ago, more or less, the same paper gave forth similar opinions with regard to the registration of midwives, but then, far from "being at one" with Mrs. Nichols, as it states in this week's number, it *then* considered the registration of *nurses* of far more importance than that of midwives. I, then, as a midwife in practice, and as one who some years ago took an active part in the promotion of the Bill, and who had then in my charge the petitions in favour of it, which were, when filled, sent into Parliament, wrote a refutation of some of the statements made in the article. Whether or not I am the "illiterate individual" alluded to in the current number I know not. Anyhow, my letter evidently found rest in the editor's waste-paper basket, and I was informed in the "Answers to Correspondents" that I "had not fully considered the question," or words to that effect. In the current number the impression is given that few obstetricians of eminence approve the Bill, and that Dr. Matthews Duncan is one who does not. This impression I wish to correct. I had the honour of taking a copy of the petition to Dr. Matthews Duncan, who most kindly gave the Matrons' Aid Society some advice as to a slight alteration in the wording of the petition, and signed it. It has been also signed by Dr. Clement Godson, no mean authority in the matter, to say nothing of Dr. J. Aveling, who from the first has been most energetic about it. The Bill also received the warm approval of the General Medical Council, in, I think, 1883, and of Lord Carlingford and Mr. Mundella, on behalf of the Privy Council. In my rejected letter I also combated the statement then made, and now repeated, that if the Bill became law an uncertified woman would not be allowed, *faute de mieux*, to assist a neighbour in her hour of need. The Bill does not enact that no woman should in an emergency do the best she can for the sufferer, but it does enact that no unqualified woman shall call herself midwife, and regularly practise as such, making engagements to attend women, and taking fees for so doing. For example, if Mrs. A. engages Mrs. B., a certificated midwife, to attend her in her confinement, and is taken suddenly ill, there is nothing in the Bill to prevent Mrs. C. doing all she can for Mrs. A. till the arrival of Mrs. B. But Mrs. C. must not on the strength of this call herself a midwife, or practise as one. It is a pity that the publication in question persistently brings forward this erroneous statement. It proves that the editor has never read the Bill. Again, the current leader sneers at the smallness of the number of midwives belonging to the Matrons' Aid Society. The writer should remember that the number of certified midwives is small compared with that of nurses. I fancy the percentage of midwives, members of the Matrons' Aid Society, will compare favourably with that of nurses who have joined the British Nursing Association. I should like to know why it need be so much more impossible to put unqualified midwives out of the field than half-trained nurses—yet this latter the British Nursing Association aims at doing? Moreover, the Bill provides that its provisions shall not come into force for six months after it becomes law, so as to give time for all uncertified midwives to qualify themselves if they wish to continue their practice. One more remark and I have done: The writer of the "leader" mourns over what the Matrons' Aid Society, or Midwives' Institute, has, in his idea, failed to do. I should like to know what *is* the great "amount of success" that the British Nurses' Association has achieved. In what does its success consist? In having held a certain number of drawing-room meetings, at which a great deal of talk took place, with but small results? And in having got half-a-crown apiece out of a few hundreds of nurses, who are now, many of them, enquiring what it was wanted for, as they have got nothing by it? This seems to onlookers *all*. The Midwives' Institute can give a better account of itself than this, despised as its efforts are by the paper referred to.

### THE PENSION FUND.

A. G. C. writes: Accept congratulations on full success of the Pension Fund with the special bonus for first thousand.

L. A. writes: I am sure all we of the nursing world feel very grateful for what you are doing for us. I only regret it was not started in my early nursing days.

C. M. M. writes: I am sure no nurse ought to be grateful enough to you gentlemen for taking so much trouble for our interest.

### CHRISTMAS DECORATIONS.

A. M. P. writes: Can any of your readers suggest any novelties in Christmas decorations for large infirmary wards? Any hints on this subject would be acceptable.

### Appointments.

[It is requested that successful candidates will send a copy of their applications and testimonials, with date of election, to THE EDITOR, The Lodge, Porchester-square, W.]

KILMARNOCK ASSOCIATION.—Miss Robin has been appointed district nurse at Kilmarnock. Miss Robin is the first nurse trained by the Scottish branch of the Queen Victoria Institute under Miss Peter. Miss Robin trained for two years at Edinburgh Royal Infirmary, for three months at Glasgow Maternity Hospital, and for six months at the Scottish Training Home.

SWANSEA HOSPITAL.—On October 10th, Miss Edith Bellars was, by a large majority, elected matron of Swansea Hospital. Miss Bellars trained at the London Hospital, and took her certificate in 1884. In 1885, Miss Bellars became night superintendent at Bristol General Hospital, which post she has held to the satisfaction of all, for over four years. She frequently took matron's duty during the latter's absence, and is well qualified to manage a staff of nurses. Miss Bellars has excellent testimonials, and takes the good wishes of all to her new post.

### Notices.

THE Harvest Festival at the Hospital for Sick Children, Great Ormond-street, will be held to-day (October 19th), at 4.45.

A COURSE of lectures on Elementary Electro-Physiology and Therapeutics are being given on Tuesday evenings at 8, at the Institute of Medical Electricity, 24A, Regent-street.

### Notes and Queries.

TO CORRESPONDENTS.—1. Questions may be written on post-cards. 2. Advertisements in disguise are inadmissible. 3. In answering a query please quote the number. 4. If a private answer is desired, a stamped addressed envelope must be forwarded.

NOTICE.—We must really ask our correspondents to be more particular in enclosing name and address. Constantly we receive queries or letters with no name attached.

### Queries.

(7) *Nursing Charts.*—Will someone tell me where to procure Dr. Jeaffreson's nursing charts? *A. E. S.*

(8) *Obstetrical Society.*—How long must I train for, and what are the fees for the London Obstetrical Society's examination? *Alice.*

(9) *To obtain Nurses.*—Kindly let me know the best place to obtain nurses for a short or long period for an infectious hospital. *A. J. R.*

(10) *Male Nurses.*—Can a man get trained as a nurse not by the Hamilton Association? *J. B.*

### Answers.

*Home for Incurables.*—Miss Lucy Hansell has seen "Kappa's" question in THE HOSPITAL for October 5, 1889, about a home for incurable and chronic diseases. She can strongly recommend All Hallow's Hospital, Ditchingham, Bungay, Norfolk. The air is bracing, not being far from the sea. Charge, 10s. a week for permanent patients.

(8) *Obstetrical Society.*—Three months' training in a lying-in hospital where good lectures are given. The fee is one guinea. Apply for particulars to Secretary, 54, Berners-street, W.

(9) *To obtain Nurses.*—We should suggest that you apply to Fitzroy House, Fitzroy-square.

(10) *Male Nurses.*—Apply to the General Nursing Institute, or to Mr. Wilson's institution. The Whitechapel Infirmary and various private hospitals also train male nurses.

### Vacancies.

THE following vacancies are announced:

### Matrons.

Royal Hospital for Children and Women, £60.
Western Fever Hospital, £100

### Nurses.

Arbroath Infirmary, £20.
Bedford Nurses' Institute, £26.
Bournemouth Nurses' Institute, £25.
Brighton Fever Hospital, £28.
Brixton and South London Nursing Institute (several).
Buckingham Nursing Home (assistant).
Carmarthen Infirmary, £23.
Frome Nurses' Home, £20.
Hitchin Nurses' Institute, £25.
Holborn Union, £25.
Jersey Nurses' Institute, £25.
Kent Nursing Institution.
Leicester Fever Hospital.
Leith Hospital, N.B., £25.
Levick Institution, Paris.
Memorial Hospital, Jarrow, £20.
Newport Infirmary, £25.
Nottingham Institution, £25.
Nurses' Home, Glossop-road, Sheffield.
Nurses' Home, Hull, £25.
Nurses' Home, Newcastle.
Nursing Institution, 96, 97, 98, Wimpole-street, London, W., Mr. Wilson has several vacancies for thoroughly trained nurses.
Parkside Asylum, £45.
Portsmouth Infectious Diseases Hospital.
Royal Albert Hospital, Devonport.
Ryde and Ventnor Nurses' Homes (three).
Samaritan Free Hospital.
Scarborough Nurses' Institute.
South Devon Hospital (two), £20.
Southport Nurses' Home.
St. George's Home, Sheffield.
St. Helena Home, N.W.
Stoke-on-Trent Home, £25.
Swansea Nursing Institute, £25.
West Bromwich District Hospital (two), £22.
Wolverhampton Nurses' Institute (several).

### Probationers.

Bath Nurses' Home.
Halifax Fever Hospital, £14.
Hull Royal Infirmary.
Ventnor Consumptive Hospital, £10.
Windsor Royal Infirmary.

### Amusements and Relaxation.

### N.B. Third Quarterly Word Competition Commenced October 5th, 1889, ends December 28th, 1889.

Three Prizes of 15s., 10s., 5s., will be given for the largest number of words derived from the words set for dissection.

Proper names, abbreviations, foreign words, words of less than four letters, and repetitions are barred; plurals, and past and present participles of verbs, are allowed. Nuttall's *Standard* dictionary only to be used.

N.B.—Word dissections must be sent in WEEKLY, arranged alphabetically, with correct total affixed.

The word for dissection for this, the *Third* week of the quarter, being "NIGHTINGALES."

### Results of First Week.

| Names. | Oct. 10th. | Totals. | Names. | Oct. 10th. | Totals. |
|---|---|---|---|---|---|
| Winifred | 18 | 18 | M. W. | 17 | 17 |
| Adelaide | 18 | 18 | Esperance | 17 | 17 |
| Nettie Vance | 12 | 12 | Sunshine | 14 | 14 |
| F. W. P. H. | 17 | 17 | Electrician | 18 | 18 |
| Amos | 17 | 17 | Reldas | 17 | 17 |
| Reyot | 17 | 17 | H. C. | 16 | 16 |
| A. S. K. | 12 | 12 | Rover | 17 | 17 |
| Ellice | 17 | 17 | Beryl | 11 | 11 |
| Neptune | 17 | 17 | Qu'appelle | 18 | 18 |
| Ecila | 17 | 17 | Hercules | 12 | 12 |
| Tinie | 17 | 17 | Lightowlers | 16 | 16 |
| Amicus | 17 | 17 | Jenny Wren | 17 | 17 |
| Juvenis | 17 | 17 | Oakenrod | 16 | 16 |
| Sister | 14 | 14 | Gypsye | 16 | 16 |
| Jumps | 17 | 17 | | | |

### Notice to Correspondents.

N.B.—All letters referring to this page which do not arrive at 139 and 140, Salisbury-court, London, E.C., by the *first post on Thursdays*, and are not addressed PRIZE EDITOR, will in future be disqualified and disregarded.

### Conditions.

I. Every paper sent in must be clearly written, and one side only must be used. All competitors must mark at the head of their papers the number of their competition.

II. Each paper must be signed by the author with his or her real name and address. A *nom de plume* may be added if the writer does not desire to be referred to by us by his real name. In the case of all prize-winners, however, the real name and address will be published.

III. All communications relating to prize competitions should be addressed to "The Prize Editor, THE HOSPITAL, 139 and 140, Salisbury-court, London, E.C." Competitors are requested not to include in letters, etc., to the Prize Editor communications on matters of other business. All letters must be fully prepaid.

IV. The Prize Editor of THE HOSPITAL is the sole judge of the competitions, and his decision is in all cases final and without appeal.

V. The Prize Editor reserves the right to publish any paper sent in, whether it receives a prize or not. He does not hold himself responsible for any MSS. sent, nor can he undertake to return rejected competitions.

Competitors can enter for *all* quarterly competitions, but no competitor may take more than *two quarterly prizes* during the year.

